# Connexin 43-Mediated Gap Junction Communication Regulates Ameloblast Differentiation *via* ERK1/2 Phosphorylation

**DOI:** 10.3389/fphys.2021.748574

**Published:** 2021-09-24

**Authors:** Aya Yamada, Keigo Yoshizaki, Masaki Ishikawa, Kan Saito, Yuta Chiba, Emiko Fukumoto, Ryoko Hino, Seira Hoshikawa, Mitsuki Chiba, Takashi Nakamura, Tsutomu Iwamoto, Satoshi Fukumoto

**Affiliations:** ^1^Division of Pediatric Dentistry, Department of Oral Health and Development Sciences, Tohoku University Graduate School of Dentistry, Sendai, Japan; ^2^Section of Orthodontics and Dentofacial Orthopedics, Division of Oral Health, Growth and Development, Faculty of Dental Science, Kyushu University, Fukuoka, Japan; ^3^The Department of Pathology and Laboratory Medicine, Perelman School of Medicine at the University of Pennsylvania, Philadelphia, PA, United States; ^4^Section of Oral Medicine for Children, Division of Oral Health, Growth and Development, Faculty of Dental Science, Kyushu University, Fukuoka, Japan; ^5^Division of Molecular Pharmacology and Cell Biophysics, Department of Oral Biology, Tohoku University Graduate School of Dentistry, Sendai, Japan; ^6^Division of Oral Health Science, Department of Pediatric Dentistry/Special Needs Dentistry, Graduate School of Medical and Dental Science, Tokyo Medical and Dental University, Tokyo, Japan

**Keywords:** connexin, gap junction, ameloblast, tooth development, dental epithelium

## Abstract

Connexin 43 (Cx43) is an integral membrane protein that forms gap junction channels. These channels mediate intercellular transport and intracellular signaling to regulate organogenesis. The human disease oculodentodigital dysplasia (ODDD) is caused by mutations in Cx43 and is characterized by skeletal, ocular, and dental abnormalities including amelogenesis imperfecta. To clarify the role of Cx43 in amelogenesis, we examined the expression and function of Cx43 in tooth development. Single-cell RNA-seq analysis and immunostaining showed that *Cx43* is highly expressed in pre-secretory ameloblasts, differentiated ameloblasts, and odontoblasts. Further, we investigated the pathogenic mechanisms of ODDD by analyzing *Cx43*-null mice. These mice developed abnormal teeth with multiple dental epithelium layers. The expression of enamel matrix proteins such as ameloblastin (Ambn), which is critical for enamel formation, was significantly reduced in *Cx43*-null mice. TGF-β1 induces *Ambn* transcription in dental epithelial cells. The induction of Ambn expression by TGF-β1 depends on the density of the cultured cells. Cell culture at low densities reduces cell–cell contact and reduces the effect of TGF-β1 on Ambn induction. When cell density was high, Ambn expression by TGF-β1 was enhanced. This induction was inhibited by the gap junction inhibitors, oleamide, and 18α-grycyrrhizic acid and was also inhibited in cells expressing Cx43 mutations (R76S and R202H). TGF-β1-mediated phosphorylation and nuclear translocation of ERK1/2, but not Smad2/3, were suppressed by gap junction inhibitors. Cx43 gap junction activity is required for TGF-β1-mediated Runx2 phosphorylation through ERK1/2, which forms complexes with Smad2/3. In addition to its gap junction activity, Cx43 may also function as a Ca^2+^ channel that regulates slow Ca^2+^ influx and ERK1/2 phosphorylation. TGF-β1 transiently increases intracellular calcium levels, and the increase in intracellular calcium over a short period was not related to the expression level of Cx43. However, long-term intracellular calcium elevation was enhanced in cells overexpressing Cx43. Our results suggest that Cx43 regulates intercellular communication through gap junction activity by modulating TGF-β1-mediated ERK signaling and enamel formation.

## Introduction

Cell–cell interactions through gap junctions are important for cell differentiation and the maintenance of cellular functions. Gap junction proteins form hexameric complexes that allow the intercellular transport of small molecules, including Ca^2+^, IP3, and cAMP (Harris, 2007; Nielsen et al., 2012). The *Gja1* gene, which encodes a typical gap junction protein, connexin 43 (Cx43), has been identified as a tumor suppressor gene (Loewenstein and Kanno, 1966, 1967; Aasen et al., 2016). This gene is important for the electrical conduction system of the heart, which may synchronize concurrent functions in cells (Verheule and Kaese, 2013; Kurtenbach et al., 2014; Macquart et al., 2018). Cx43 is also involved in the human disease oculodentodigital dysplasia (ODDD; Paznekas et al., 2003, 2009; Laird, 2014), a disorder characterized by congenital missing teeth, microdontia, enamel hypoplasia, syndactyly, osteodysplasia, and craniofacial deformities (Judisch et al., 1979; Paznekas et al., 2003). Since the epithelial–mesenchymal interaction in tooth development is similar to interactions in the apical ectodermal ridge region during limb development, disorders presenting with simultaneous malformations of the teeth and fingers are frequently observed in human genetic diseases.

Regarding the role of Cx43 in tooth development, immunostaining for Cx43 has been reported in tooth germ layers, particularly in the inner enamel epithelium and ameloblasts (Al-Ansari et al., 2018; Nakamura et al., 2020). However, an analysis of teeth in Cx43 gene-deficient mice has not been sufficiently performed because the mice die during the fetal period due to heart and lung malformations (Reaume et al., 1995; Huang et al., 1998; Nagata et al., 2009). In *Gja1Jrt/*+ mice with mutations in the *Cx43* gene, the effect of *Cx43* gene abnormalities on tooth formation was reported. Since these mice are viable, unlike *Cx43* gene-deficient mice, it was possible to analyze tooth formation after eruption. In this mouse, enamel hypoplasia and abnormalities in the arrangement of ameloblasts were observed (Flenniken et al., 2005; Toth et al., 2010). Furthermore, in mice in which the *Cx43* gene was ablated selectively in DMP1-expressing cells, a decrease in enamel formation and reduced mineral density were observed. This evidence suggests that Cx43 may be involved in the regulation of mineral transport in mature ameloblasts. However, these mice were analyzed mainly using histological examination, which does not fully clarify the underlying molecular function. Thus, the role of Cx43 in ameloblasts and its effects on tooth development are still unknown.

We identified tooth-specific molecules and analyzed their functions to clarify the differentiation mechanism of ameloblasts. Ameloblasts express the enamel matrix, which is a cell-specific extracellular matrix. Enamel is formed through the decomposition and absorption of the substrates. AMBN-deficient mice exhibit severe enamel hypoplasia and the formation of odontogenic tumors (Fukumoto et al., 2004). In addition, various transcription factors are involved in AMBN expression. Among them, Sp6/Epfn is strongly expressed in the inner enamel epithelium and ameloblasts. Sp6/epiprofin-deficient mice have an increased number of tooth germs, and teeth are formed without enamel (Nakamura et al., 2008, 2017). Sox21 is also involved in the regulation of *Ambn* expression and in the fate determination of invaginated epithelial cells. In Sox21-deficient mice, hair develops from the dental epithelium and epithelial–mesenchymal transition occurs in some tooth cells (Saito et al., 2020). Furthermore, Panx3, which is a gap junction strongly expressed in teeth, is expressed in odontoblast progenitor cells, is involved in dentin formation, and plays an important role in bone formation (Iwamoto et al., 2010, 2017; Ishikawa et al., 2011). Panx3 induces Cx43 expression and plays an important role in gap junction formation and osteoblast differentiation in bone (Ishikawa et al., 2016). Specifically, Panx3 and Cx43 regulate bone formation through their coordinated and continuous spatiotemporal expression. While Cx43 is expressed in ameloblasts, Panx3 is not, suggesting that hard tissue formation in ameloblasts may be regulated by a mechanism different from that of bone.

The aim of this study was to clarify the function of Cx43 in tooth development and to elucidate the common system involved in ectodermal organogenesis.

## Materials and Methods

### Reagents

Anti-Cx43 and Runx2 antibodies were obtained from Santa Cruz Biotechnology. Anti-ERK1/2, phospho-ERK1/2, Smad2/3, phospho-Smad2/3(465/467), SAPK, phospho-SAPK, p38, phospho-p38, and HPRP-conjugated anti-rabbit IgG were obtained from Cell Signaling Technology. Anti-phospho-serine antibodies were purchased from Sigma-Aldrich. The anti-AMBN antibody has been previously described (Fukumoto et al., 2004). Alexa-488 or 594 conjugated anti-rabbit IgG was purchased from Molecular Probes. 18α-glycyrrhetinic acid (18α-GA), oleamide, adenophostin-A, and 2-APB were obtained from Sigma-Aldrich. PD98059 was obtained from Cell Signaling Technology. Fura-2AM was obtained from Invitrogen. TGF-β1, BMP2, and BMP4 were obtained from R&D Systems. Briefly, the pEF6/Cx43 vector was constructed by cloning the coding sequence of mouse Cx43 cDNA into the pEF6/V5-His TOPO vector (Invitrogen). Cx43 expression vectors carrying R76S or R202H mutations were prepared using a Quick Change Site-Directed Mutagenesis Kit (Stratagene). siRNA for Cx43 was purchased from Invitrogen.

### Single-Cell Preparation, Library Construction, and Sequencing

To prepare single-cell RNA sequencing (scRNA-seq) samples, we dissected the incisors from littermates of Krt14-RFP mice, as previously described (Chiba et al., 2020; Wang et al., 2020). Single cells were captured and a single-cell library was constructed using a 10x Chromium Single-Cell 3’ Reagent Kit (10x Genomics, San Francisco, CA, USA). Then, libraries were sequenced on a NextSeq 500 sequencer (Illumina, San Francisco, CA, USA). The experiments were independently performed twice. Data processing was performed using the 10x Genomics workflow (Zheng et al., 2017). Demultiplexing, barcode assignment, and unique molecular identifier (UMI) quantification was performed using the Cell Ranger Single-Cell Software Suite (10x Genomics). The datasets generated for this study can be found in the NCBI GEO: GSE146855.

### Semiquntitive and Real-Time PCR

Total RNA was isolated using TRIzol reagent (Invitrogen). Then, total RNA (2μg) was reverse-transcribed into cDNA in 20μl 1× first-strand buffer containing 0.5μg oligo(dT) primers, 500μM dNTP, and 200units of SuperScript III (Invitrogen). PCR was performed in 25μl 1× first PCR buffer containing 2μl reverse transcription products, 1 unit of Ex TaqDNA polymerase (Takara, Japan), 200μM dNTP, and 0.4μM of the primer pair. The specific forward and reverse primers used for PCR were as follows: *Cx43*; 5'-GAGTCAGCTTGGGGTGATGAACAG-3' and 5'-AGCAGGAAGGCCACCTCGAAGACAGAC-3'; *Ambn*; 5'-GCGTTTCCAAGAGCCCTGATAAC-3' and 5'-AAGAAGCAGTGTCACATTTCCTGG-3'; *Enam*; 5'-GTGAGGAAAAATACTCCATATTCTGG-3' and 5'-GTTGAAGCGATCCCTAAGCCTGAAGCAG-3'; *Amel*; 5'-ATTCCACCCCAGTCTCATCAG-3' and 5'-CCACTTCGGTTCTCTCATTTTCTG-3'; *Bmp2*, 5'-GGGACCCGCTGTCTTCTAGT-3' and 5'-TCAACTCAAATTCGCTGAGGAC-3'; *Bmp4*, 5'-ACTGCCGCAGCTTCTCTGAG-3' and 5'-TTCTCCAGATGTTCTTCGTG-3'; *Hprt*; 5'-GCGTCGTGATTAGCGATGATGA-3' and 5'-GTCAAGGGCATATCCAACAACA-3'; *Runx2*; 5'-GAGGCCGCCGCACGACAACCG-3' and 5'-CTCCGGCCCACAAATCTCAGA-3'. The PCR products were separated on 1.5% agarose gels. Real-time PCR amplification was performed using primers with SYBR Green PCR master mix and a TaqMan 7700 Sequencer (Applied Biosystems). PCR was performed for 40cycles of 95°C for 1min, 60°C for 1min, and 72°C for 1min.

### Preparation of Tissue Samples and Immunostaining

All animal experiments were approved by the ethics committee of Kyushu University Animal Experiment Center, and all procedures were performed in accordance with the relevant guidelines and regulations. The ICR mice (SLC) and Cx43-null mutants (Jackson Labs) used for experiments were previously described (Nagata et al., 2009; Ishikawa et al., 2016; Yamada et al., 2016). The tooth germ was dissected at postnatal day 3 (P3), and brain, lung, heart, liver, kidney, testis, and skin were obtained from 8-week-old ICR mice. Incisor presecretory (PS), secretory (S), early maturation (EM), and late maturation (LM) samples were dissected from the lower incisors of 8-week-old ICR mice. Molar samples were collected from embryonic day 13.5 (E13.5) to P7 ICR mice and newborn *Cx43*^−/−^ mice. The dental epithelium and dental mesenchyme of E17.5 and P1 molars were treated with 0.1% collagenase, 0.05% trypsin, and 0.5mM EDTA and separated under a microscope. For histological analysis, P0 mouse heads were dissected and fixed with 4% paraformaldehyde in phosphate-buffered saline overnight at 4°C. Tissues were embedded in OCT compound (Sakura Finetechnical Co.) cut at 8μm thickness with a cryostat (2,800 Frigocut, Leica, Inc.) for frozen sectioning. Additional fixed tissue samples were cleared with xylene, dehydrated with a graded ethanol series, embedded in Paraplast paraffin (Oxford Laboratories), and sectioned at 10μm with a microtome (RM2155, Leica, Inc.). For morphological analysis of the molars and incisors, sections were stained with Harris hematoxylin (Sigma) and eosin Y (Sigma). Immunohistochemistry was performed on sections, which were blocked in 1% bovine serum albumin/phosphate-buffered saline for 1h and incubated with primary antibody. Primary antibodies were detected using AlexaFluor 488- or 594-conjugated secondary antibodies (Molecular Probes). Nuclei were stained with Vectashield-DAPI hard set (Vector). Tissue and cell samples for immunohistochemistry were examined using a fluorescence microscope (Biozero-8,000; Keyence, Japan).

### Cell Culture and Transfection

Dental epithelial cell cultures were derived from molars dissected from newborn mice. The molars were treated with 0.1% collagenase, 0.05% trypsin, and 0.5mM EDTA for 10min to separate the dental epithelium from the mesenchyme. The separated dental epithelium was treated with 0.1% collagenase, 0.05% trypsin, and 0.5mM EDTA for 15min. The cells were dispersed into culture wells by repeated withdrawal and release using a pipette. Dental epithelial cells were then selected after 7days of culture in keratinocyte-serum-free medium (Invitrogen) supplemented with epidermal growth factor and bovine pituitary extract, which removed contaminating mesenchymal cells. Cells were then detached with 0.05% EDTA, washed with DME containing 0.1% bovine serum albumin, resuspended to a concentration of 1.0×10^5^/ml, and used for cell adhesion assays. SF2 cells from rat dental epithelium were maintained in Dulbecco’s modified Eagle’s medium/F-12 medium supplemented with 10% fetal bovine serum (Arakaki et al., 2012). All cells were cultured with 1% penicillin and streptomycin (Invitrogen) at 37°C in a humidified atmosphere containing 5% CO_2_. SF2 cells were transiently transfected with Cx43, Cx43-R76S, or Cx43-R202H expression plasmids or Cx43 siRNA using Lipofectamine 2000 and Oligofectamine (Invitrogen), respectively.

### Western Blotting

Cells were washed twice with 1mM ice-cold sodium orthovanadate (Sigma), lysed with Nonidet P-40 buffer supplemented with protease inhibitors at 4°C for 10min, and centrifuged. Then, the supernatants were transferred to a fresh tube. After boiling for 10min, the proteins were separated by 12% SDS-PAGE and analyzed by Western blotting. Blots were probed using anti-ERK1/2, phospho-ERK1/2, Smad2/3, phospho-Smad2/3(465/467), SAPK, phospho-SAPK, p38, phospgo-p38, phospho-serine, and Runx2 with horseradish peroxidase-linked anti-rabbit secondary antibody (Cell Signaling). Proteins were detected with ECL Western blotting detection reagents (Amersham Biosciences) and exposed to X-ray film or imaged using a LAS-4000 system (Fuji Film, Japan). For immunoprecipitation assays, cells were seeded in 10-cm dishes at a density of 1×10^6^/dish and cultured for 1week and then harvested for protein extraction. Immunoprecipitation was carried out using a Dynabeads Protein G kit (Life Technologies). Anti-Runx2 antibodies were fused to protein G magnetic beads and incubated with the samples for 1h at 4°C. The complex was eluted and denatured with NuPAGE LDS sample buffer (Life Technologies) supplemented with 1% 2-mercaptoethanol. The samples were analyzed using Western blotting.

### Intracellular Calcium Measurement

SF2 cells were grown in a 96-well plate for 3days and then incubated with 5μM Fura-2AM (Invitrogen) prepared in HBSS for 45min at 37°C in 5% CO_2_. After 3days, the SF2 cells were transiently transfected with either Cx43 or control siRNA. The Ca^2+^ transients were recorded as the 340/380nm ratio (R) of the resulting 510-nm emission using a plate reader (Mithras LB 940; Berthold Technologies). For stimulation, TGF-β1 was automatically injected into cells using the Mithras 940 instrument. For inhibition experiments, cells were incubated for 30min before analysis with wither 100μM 2-APB (to block IP3R) or 18α-Gly (to block gap junctions). After 3days of transfection, the intracellular calcium ([Ca^2+^]_i_) was measured. The [Ca^2+^]_i_ levels were calculated as described previously (Grynkiewicz et al., 1985; Ishikawa et al., 2011) using the equation [Ca^2+^]_i_=K_d_ (R−R_min_)/(R_max_−R)(F^380^_max_/F^380^_min_), where R_min_ is the ratio at zero Ca^2+^, R_max_ is the ratio when Fura-2 is completely saturated with Ca^2+^, F^380^_min_ is the fluorescence at 380nm for zero Ca^2+^, and F^380^_max_ is the fluorescence at saturating Ca^2+^ and K_d_=224nM.

### Data Analysis

Statistical differences between two groups of data were analyzed using Student’s *t*-test. One-way ANOVA was used to analyze multiple groups. Differences were considered statistically significant at *p*<0.05.

## Results

### Expression of *Cx43* in Tooth Germ

**Figure 1:**
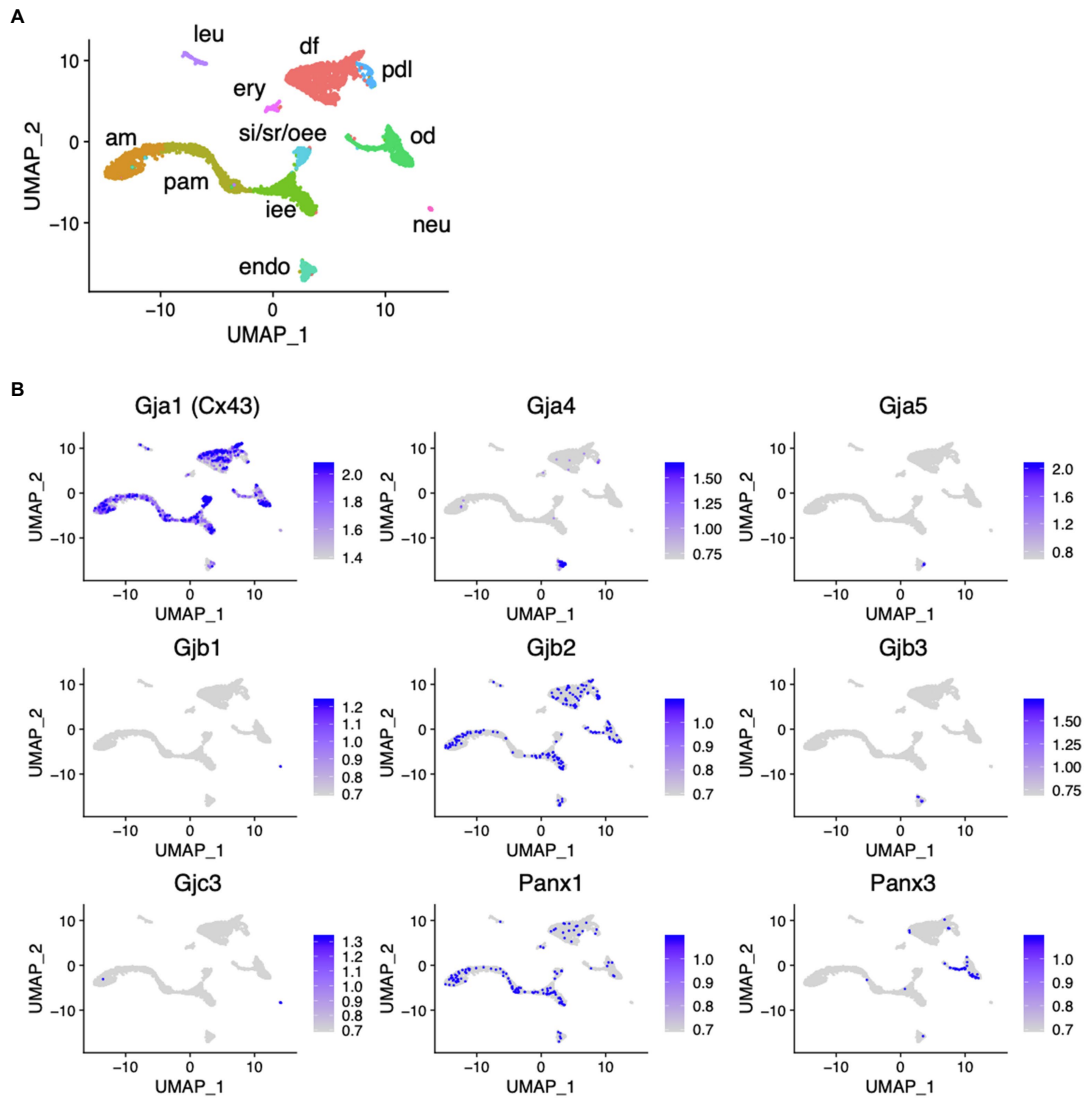
Expression pattern of connexin family members during tooth development. **(A)** Differential expression of analysis of cell-type marker genes using uniform manifold approximation and projection (UMAP) visualization of single-cell RNA-seq libraries from postnatal (P) day P7 Krt14-RFP mice incisors (Chiba et al., 2020). am, ameloblast; Pam, pre-ameloblast; iee, inner enamel epithelium; si/sr/oee, stratum intermedium, stellate reticulum, and outer enamel epithelium; od, odontblast; df, dental follicle; pdl, periodontal ligament; leu, leukocyte; ery, erythrocyte; neu, neural cell; encoendothelium. **(B)** Differential gene expression analysis of connexin family members detected in single-cell RNA-seq libraries from P7 Krt14-RFP mice incisors.

scRNA-seq was performed using dissociated cells from Krt14-RFP mouse incisors to obtain single-cell gene expression profiles as described previously (Figure 1A; Chiba et al., 2020). In the incisors, well-distinguished cell types were observed: inner enamel epithelium (iee), pre-ameloblasts (pam), ameloblasts (am), outer enamel epithelium (oee), stratum intermedium (st), stellate reticulum (sr) as dental epithelial cells, odontoblasts (od), dental pulp cells (dp), dental follicles (df), and periodontal ligament cells (dl) as dental mesenchymal cells. *Gja1* (Cx43), *Gjb2*, and *Panx1* were expressed in the dental epithelium. *Cx43*, *Gjb2*, and *Panx3* were expressed in the dental mesenchyme (Figure 1B).

**Figure 2:**
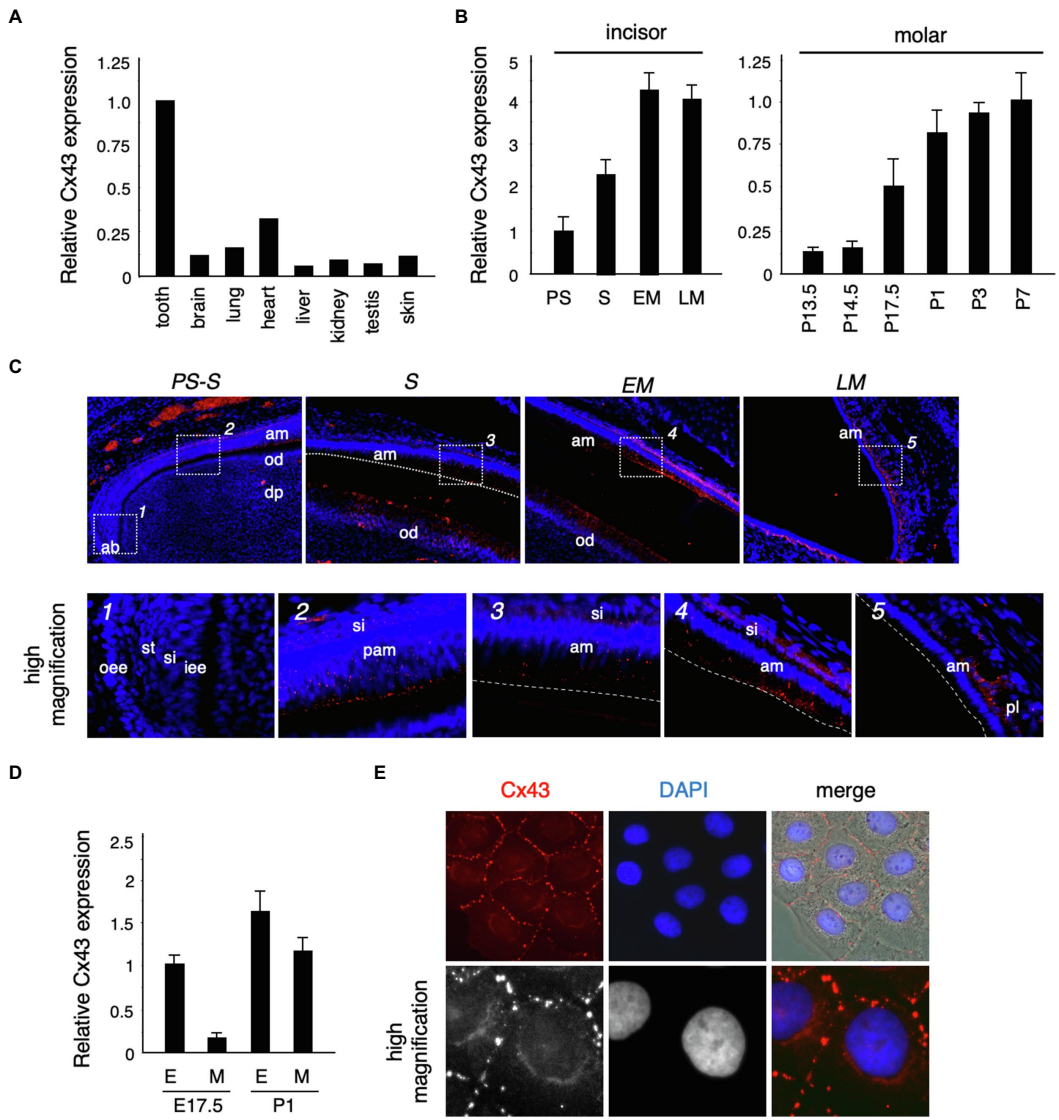
Connexin 43 (*Cx43*) expression in tooth germ development. **(A)** Expression of *Cx43* mRNA in various tissues. **(B)** Expression of *Cx43* mRNA by ameloblasts in the presecretory (PS), secretory (S), early maturation (EM), and late maturation (LM) stages of incisor development. Expression of *Cx43* mRNA from embryonic day 13.5 (E13.5) to postnatal day 7 (P7) in tooth germs from whole molars. **(C)** Localization of Cx43 in developing upper incisors. A high-magnification image of the area indicated by a dotted line is shown in the lower panel. **(D)** Expression of Cx43 mRNA in separated epithelial (E) and mesenchymal (M) tooth germs. **(E)** Localization of Cx43 in cultured SF2 cells. Cx43 (red), DAPI (blue), and the merged image. High-magnification images are shown in the lower panel. ab, apical bud; am, ameloblast; od, odontoblast; dp, dental pulp; oee, outer enamel epithelum; st, stellate reticulum; si, stratum intermedium; iee, inner enamel epithelium; pam, pre-ameloblast; and pl, papillary layer.

To confirm the expression of *Cx43* in the tooth germ, we performed real-time PCR. *Cx43* was highly expressed in the tooth germ compared to expression in other tissues (Figure 2A). In the incisors, *Cx43* expression increased as differentiation progressed, and was located in the ameloblast and odontoblast layers during the secretory stage (Figures 2B,C). In molars, the enamel epithelium differentiates into ameloblasts around birth, when it begins to secrete the enamel matrix. *Cx43* expression intensified in the dental epithelium and mesenchyme after birth (Figures 2B,D). Further, Cx43 protein localized at the cell–cell interface of cultured dental epithelial cells and formed gap junctions (Figure 2E). These observations suggest that Cx43 may regulate the differentiation of ameloblasts and odontoblasts during tooth development.

### Disorganization of Ameloblasts and Decreased Ameloblastin Expression in *Cx43* Null Mice

**Figure 3:**
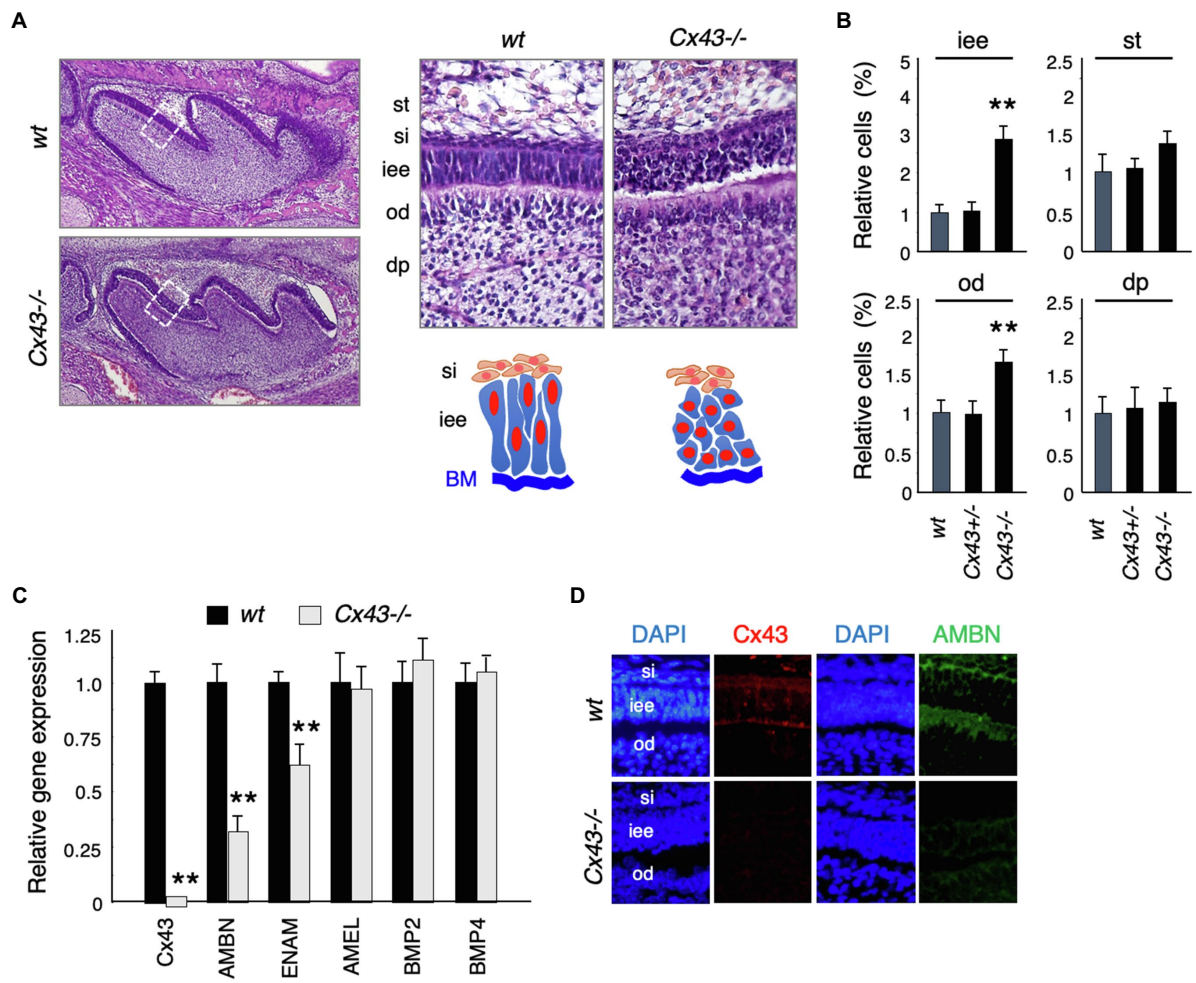
Tooth phenotypes in *Cx43*-deficient mice. **(A)** Hematoxylin staining of upper first molars in *wt* and *Cx43*^−/−^ mice, and high-magnification images and scheme of dental epithelial cells in newborn *wt* and *Cx43*^−/−^ mice. st, stellate reticulum; si, stratum intermedium; iee, inner enamel epithelium; od, odontoblast; dp, dental papilla. **(B)** Relative numbers of each cell types in *wt*, *Cx43*^+/−^, and *Cx43*^−/−^ mice. *n*−5, ^**^p<0.01. **(C)** Expression of *Cx43*, enamel matrix proteins, and BMPs in first molar tooth germs in P3 *WT* and *Cx43*^−/−^ mice. *Ambn*, ameloblastin; *Enam*, enamelin; *Amel*, amelogenin. *n*−4, ^**^p<0.01. **(D)** Localization of Cx43 (red) and Ambn (green) in *wt* and *Cx43*^−/−^ molars.

*Cx43*-null mice (*Cx43*^−/−^) showed cyanosis and died after birth due to malformations of the heart and lungs (Reaume et al., 1995; Huang et al., 1998; Nagata et al., 2009). No significant changes were noted in tooth size or shape in *Cx43*^−/−^ mice (Figure 3A), but the inner enamel epithelium (iee) lost its polarity and formed multiple layers (Figure 3A). The number of inner enamel epithelial cells and odontoblasts (od) increased, but neither stratum intermedium (st) nor stellate reticulum (si) cells were observed (Figure 3B). The expression of BMP-2 and -4, which are involved in ameloblast differentiation, was unchanged in the teeth of *Cx43*^−/−^ mice (Figure 3C). Enamel matrix proteins such as Ambn and Enam decreased significantly in *Cx43*^−/−^ teeth (Figure 3C). Further, immunostaining showed that Ambn protein expression was decreased in the *Cx43*^−/−^ tooth germ (Figure 3D). These mice died shortly after birth, so it was not possible to verify whether amelogenesis occurs in the absence of Cx43. In the case of postnatal lethality, it is also possible to observe enamel formation by transplantation of tooth germs under the renal capsule. However, enamel hypoplasia was observed in mice with a partial genetic abnormality of Cx43. The ameloblast disorganization and the decreased expression of the enamel matrix we observed are consistent with previous reports.

### TGF-β1-Induced Meloblastin Expression Is Regulated by Gap Junctional Communication

**Figure 4:**
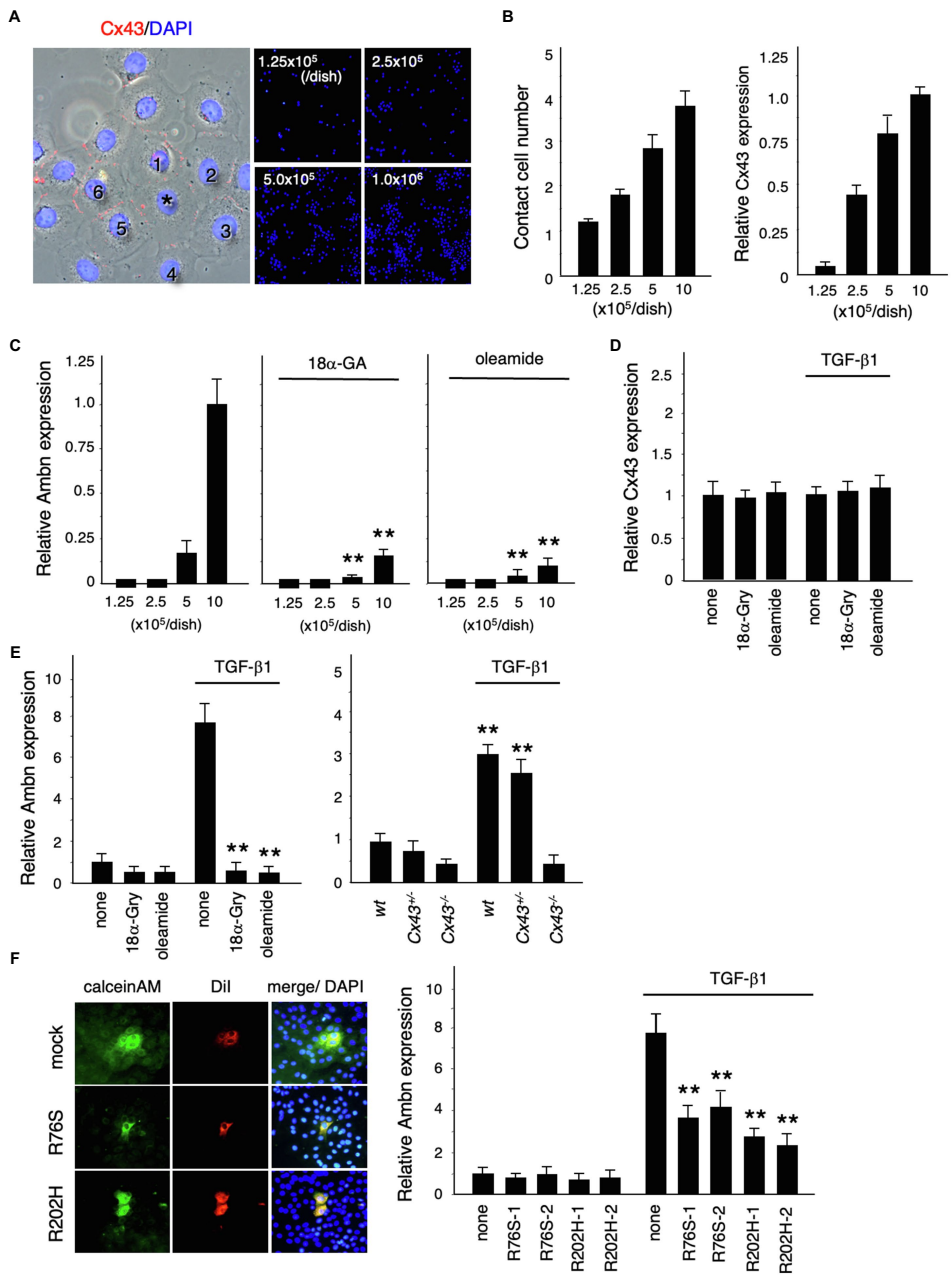
Regulation of Ambn expression in dental epithelial cells by gap junction communication. **(A)** Immunostaining of Cx43 (red) and nuclear staining with DAPI (blue) in cultured dental epithelial cells. The cell marked with an asterisk contacts six cells *via* gap junctions. **(B)** The average number of contact cells at each cell density is shown in the left panel. Expression of *Cx43* in cultured dental epithelial cells at each cell density is shown in the right panel. **(C)** Expression of Ambn at each cell density with or without the gap junction inhibitors 18α-Glycyrrhetinic acid (18α-GA) and oleamide. **(D)** Effect of gap junction inhibitors on *Cx43* after 48h of TGF-β1 stimulation (1ng/ml). **(E)** Effect of the gap junction inhibitors 18α-Gly and oleamide on Ambn expression (left panel) or on *Cx43*^−/−^ cells (right panel). **(F)** Gap junction activity was measured by transfecting dental epithelial cells with a Cx43 expression vector carrying R76S or R202H mutations. CalceinAM and DiI were injected into the center of cell. Ambn expression in cells stably transfected with Cx43 expression vectors harboring R76S or R202H mutations, which are found in human oculodentodigital dysplasia (ODDD) patients, after stimulation with TGF-β1 is shown in the right panel. ^**^p<0.01.

We investigated the role of Cx43 in dental epithelial cell proliferation and differentiation by examining the expression of Ambn in dental epithelial cells cultured at different cell densities (Figure 4A). Cx43 expression increased as the number of cells in contact increased (Figure 4B), but Ambn was not induced until a certain number of cells were in contact (Figure 4C). Ambn expression at a higher cell density was almost completely inhibited in the presence of the gap junction inhibitors (18α-GA) and oleamide (Figure 4C). However, Cx43 expression was not inhibited by gap junction inhibitors (Figure 4D). These results suggest that Cx43 gap junction activity is involved in the regulation of Ambn expression during amelogenesis.

TGF-β1 induced the expression of Ambn, which is involved in amelogenesis (Figure 4E). Additionally, TGF-β1 induced ERK1/2 phosphorylation and inhibited the proliferation of dental epithelial cells in BrdU incorporation assays (data not shown). In mammary gland epithelial cells, Cx43 expression is induced by TGF-β1 *via* the p38 and PI3 kinase/Akt pathways (Tacheau et al., 2008). However, Cx43 expression in dental epithelial cells was not affected by TGF-β1 (Figure 4D), with or without gap junction inhibitors, whereas gap junction inhibitors significantly reduced TGF-β1-induced Ambn expression (Figure 4E). Furthermore, we examined the expression of Ambn induced by TGF-β1 in cultured dental epithelial cells from *Cx43*^−/−^ mice. In *Cx43*^−/−^-derived cells, TGF-β1-induced expression of Ambn was suppressed, similar to gap junction inhibitors (Figure 4E). These findings suggest that the presence of gap junctions is important for TGF-β1-mediated Ambn expression. We also tested whether Cx43 activity was required for Ambn expression by transfecting dental epithelial cells with an expression vector for Cx43 carrying R76S or R202H mutations, as identified in patients with ODDD. In these cells, gap junction activity was reduced, as shown by dye transfer analysis (Figure 4F), and TGF-β1-induced Ambn expression was suppressed. The inhibitory effect on Ambn expression was less profound than that observed using gap junction inhibitors (Figure 4F), which is likely due to the presence of endogenous Cx43. An almost complete lack of Ambn induction by TGF-β1 was observed in a primary culture of *Cx43*^−/−^ dental epithelial cells (Figure 4E). These results suggest that Cx43 gap junction activity is essential for TGF-β1-mediated expression of Ambn and dental epithelial differentiation.

### ERK Phosphorylation and Nuclear Translocation Are Decreased by Gap Junction Inhibitors

**Figure 5:**
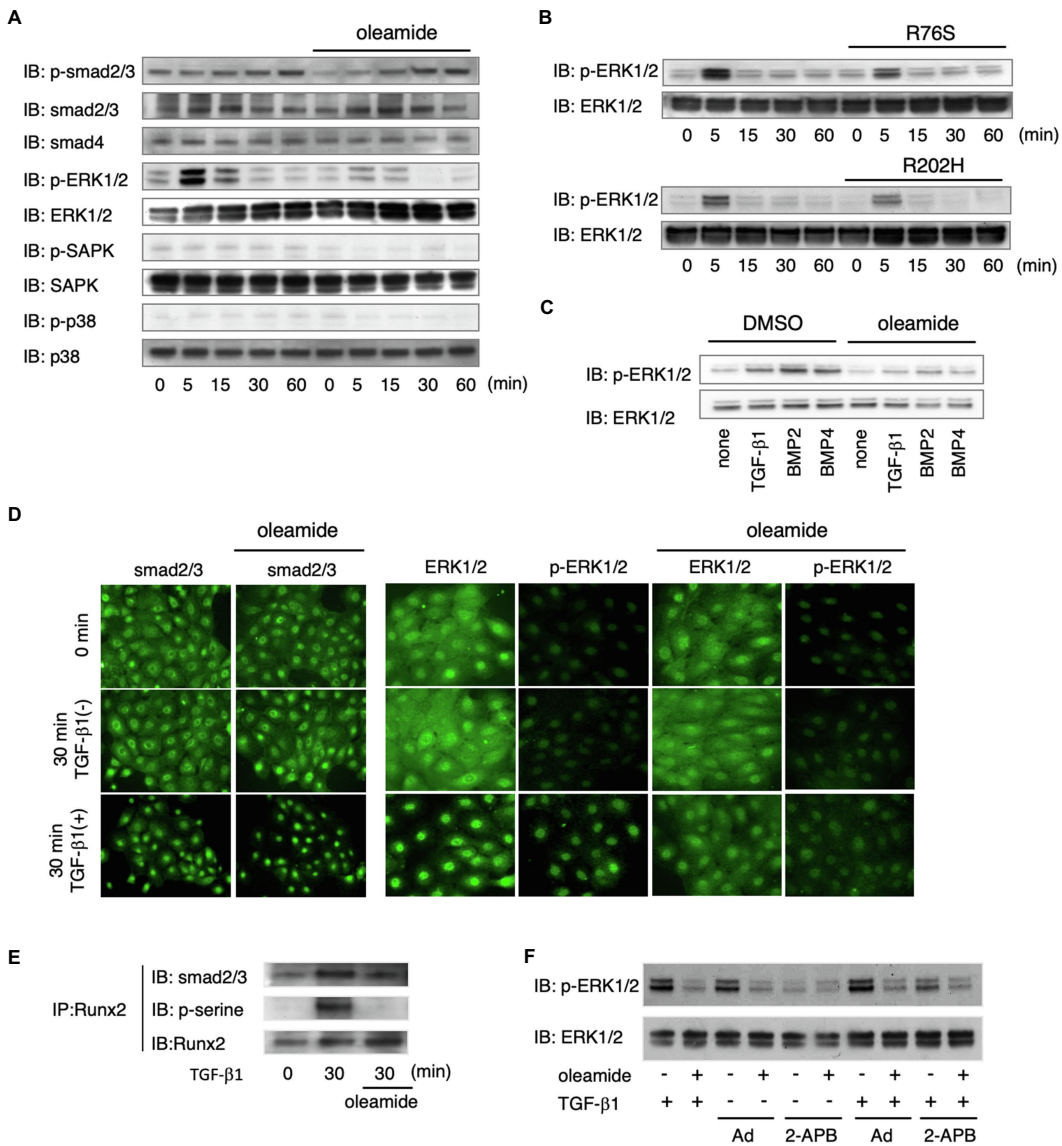
ERK1/2 phosphorylation is regulated by Cx43-mediated gap junction communication. **(A)** Phosphorylation of Smad2/3, ERK1/2, and p38 after stimulation by TGF-β1 with or without oleamide. *n*=5. **(B)** Phosphorylation of ERK1/2 after stimulation by TGF-β1 after transfection with expression vectors for Cx43 mutants (R76S or R202H). *n*=5. **(C)** Phosphorylation of ERK1/2 after stimulation by TGF-β1, BMP2, and BMP4 with or without oleamide. *n*=5. **(D)** Localization of Smad2/3 after stimulation by TGF-β1 with or without oleamide. *n*=5. Localization of ERK1/2 and phospho-ERK1/2 after stimulation by TGF-β1 with or without oleamide. *n*=5. **(E)** Immunoprecipitation of Runx2 in TGF-β1-stimulated dental epithelial cells. TGF-β1 induced Runx2 phosphorylation and caused the association of Runx2 with Smad2/3. Phosphorylation of serine residues of Runx2 and binding with Smad2/3 were inhibited by oleamide. *n*=5. **(F)** Phosphorylation of ERK1/2 in the presence of the IP3R agonist, adenophostin-A (Ad), or the IP3R antagonist, 2-APB, with or without TGF-β1 or oleamide.

We investigated the mechanism of Cx43 in TGF-β1-induced Ambn expression by analyzing TGF-β1 signaling pathways using gap junction inhibitors and Cx43 mutants. TGF-β1-induced phosphorylation of Smad2/3 was not affected by gap junction inhibitors (Figure 5A), and Smad4 levels remained unchanged (Figure 5A). In non-Smad TGF-β1 pathways, the phosphorylation of ERK1/2, but not SAPK/JNK and p38, was almost completely blocked by gap junction inhibitors (Figure 5A). In dental epithelial cells overexpressing the non-functional R76S- or R202H-Cx43 mutants, TGF-β1-stimulated ERK1/2 phosphorylation was reduced compared with that in wild-type Cx43-overexpressing cells (Figure 5B). Phosphorylated Smad2/3 forms a complex with Smad4 and translocates into the nucleus after TGF-β1 stimulation (Schmierer and Hill, 2007). In SF2 cells, this translocation was not affected by the gap junction inhibitor, oleamide (Figure 5C). TGF-β1 stimulation resulted in the translocation of most of the ERK1/2 into the nucleus. However, oleamide inhibited the nuclear translocation of ERK1/2 (Figure 5C). These results indicate that blocking gap junction activity inhibits the phosphorylation and nuclear translocation of ERK1/2. A similar regulation of ERK1/2 phosphorylation was observed following BMP2 and BMP4 stimulation (Figure 5B). Furthermore, this phenomenon was observed in dental epithelial cells and in primary cultures of calvarial osteoblasts and osteoblastic MC3T3-E1 cells (data not shown).

The translocation of phosphorylated ERK1/2 participates in the phosphorylation of Runx2, a transcription factor essential for osteogenesis, and consequently regulates the transcription of osteocalcin (Ge et al., 2007). ERK1/2 is also involved in interactions between cells and the extracellular matrix, hormones, and growth factor signals, and increases its own activity (Keshet and Seger, 2010; Guo et al., 2020). Therefore, we investigated the role of ERK1/2 in Runx2 phosphorylation during TGF-β1-mediated differentiation of dental epithelial cells. Runx2 was phosphorylated by TGF-β1, and this phosphorylation was blocked by oleamide (Figure 5E). These results suggest that Runx2 phosphorylation occurs through the TGF-β1-Cx43-ERK1/2 pathway. Immunoprecipitation analysis revealed that Smad2/3 was associated with Runx2 when stimulated by TGF-β1 in a manner similar to Smad1/Runx2 interactions during BMP-2 stimulation (Selvamurugan et al., 2004). However, the association of Smad2/3 with Runx2 was significantly reduced in the presence of oleamide, most likely due to reduced Runx2 phosphorylation (Figure 5D). Cx43 is required for growth factor-stimulated ERK1/2 phosphorylation. However, the mechanism by which Cx43 gap junction activity regulates ERK1/2 phosphorylation is not yet clear. Gap junction channels allow the transfer of IP3 and Ca^2+^ between cells. IP3 binds to its receptors (IP3Rs) in the endoplasmic reticulum (ER) membrane and promotes Ca^2+^ release from the ER (Mikoshiba, 2007), thereby increasing intracellular Ca^2+^. Ca^2+^ efflux from the ER *via* the IP3R Ca^2+^ channel is essential for the conversion of Ras-GDP to its GTP form, which is upstream of ERK1/2 (Pérez-Sala and Rebollo, 1999). We explored the involvement of the IP3/IP3R channel in the regulation of ERK1/2 phosphorylation through gap junctions by examining the effects of an IP3R agonist and antagonist on ERK1/2 phosphorylation. When dental epithelial cells were treated with the IP3R agonist adenophostin-A (Ad), which stimulates the release of Ca^2+^ from the ER, ERK1/2 phosphorylation was induced by TGF-β1 stimulation (Figure 5F). In contrast, treatment with the IP3R antagonist 2-APB inhibited ERK1/2 phosphorylation induced by TGF-β1 (Figure 5F). The phosphorylation of ERK1/2 induced by adenophostin-A was blocked by oleamide (Figure 5F). These results suggest that IP3R is involved in TGF-β1-induced ERK1/2 phosphorylation.

### Late-Phase Intracellular Calcium Level Regulated by Cx43

**Figure 6:**
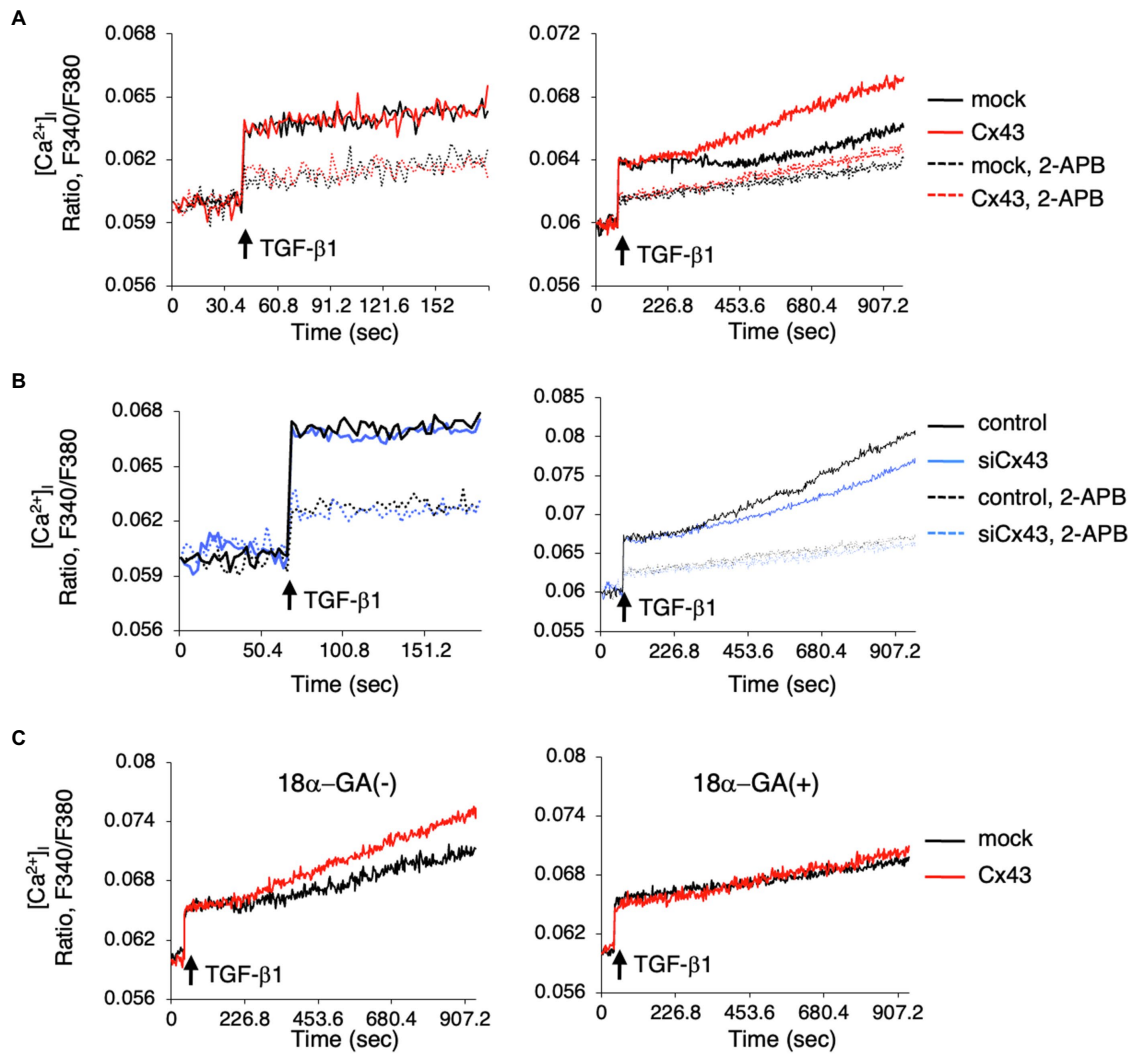
Intracellular Ca^2+^ release is induced by Cx43-containing gap junctions localized in the cell membrane. **(A)** Cells were incubated with Fura2 (10μM) with or without 2-APB (100μM). TGF-β1 induced [Ca^2+^]_i_ levels were not changed in Cx43-transfected cells compared to control cells over a short period. However, [Ca^2+^]_i_ levels were increased in Cx43-overexpressing cells after 180s compared with non-transfected cells. **(B)** [Ca^2+^]_i_ level after stimulation by TGF-β1 in Cx43 siRNA transfected cells with or without 2-APB for a short period (left panel) or a long time (right panel). **(C)** [Ca^2+^]_i_ levels after stimulation by TGF-β1 in Cx43-transfected cells with or without 18α-GA for a long period.

**Figure 7:**
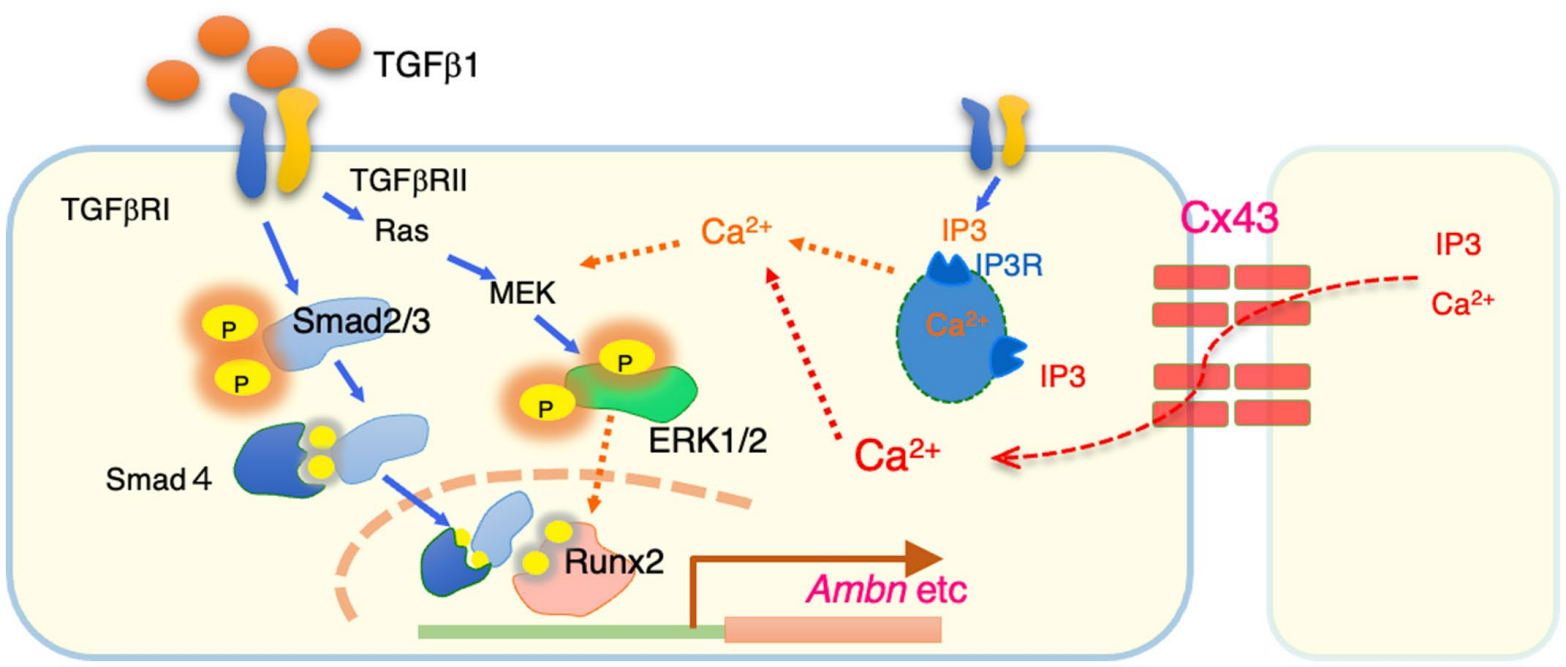
A summary of intracellular signals regulated by Cx43. Cx43 exists between cells and allows to permeate calcium ions from neighboring cells. The resulting increase in intracellular calsium promotes phosphorylation of ERK1/2 by TGF-b1. Phosphorylated ERK1/2 is thought to bind to the Smad complex in the nucleus and regulate the transcription such as Ambn.

Since intracellular calcium levels may be involved in ERK phosphorylation, we investigated whether intracellular Ca^2+^ dynamics are regulated by Cx43. We examined the role of Cx43 in maintaining intracellular Ca^2+^ levels by comparing the [Ca^2+^]_i_ levels after TGF-β1 stimulation in Cx43-overexpressing and control dental epithelial cells. The [Ca^2+^]_i_ level was increased by TGF-β1 stimulation and reduced by 2-APB (Figure 6A). No differences were observed in ER Ca^2+^ channel activity between Cx43-overexpressing and control cells within 120s (Figure 6A, left). However, the [Ca^2+^]_i_ level after 180s gradually increased in Cx43 expressing cells, with or without 2-APB, compared to control cells (Figure 6A right). Treatment with Cx43 siRNA and the gap junction inhibitor 2-APB inhibited the late phase [Ca^2+^]_i_ increase (Figure 6B). A similar phenomenon was observed using 18α-GA (Figure 6C), indicating that this late phase increase may be due to gap junctions, but not the ER channel. These results suggest that Cx43 may allow slow Ca^2+^ influx *via* gap junctions, but is not involved in the early phase [Ca^2+^]_i_ increase from the ER channel. Additionally, these results suggest that Cx43 regulates ERK1/2 phosphorylation (Figure 7).

In this study, we showed that Cx43 expression is induced in the dental epithelium when the cells are in contact with each other. Our results suggest that Cx43 gap junctions are critical for the maintenance of polarity and differentiation of dental epithelial cells. Cx43 gap junctions are also required for TGF-β1- and BMP-induced expression of enamel matrix proteins, especially Ambn. This gene induction is mediated by ERK1/2 activation *via* Cx43 gap junction activity (Figure 7). These findings provide mechanistic insights into the development of dental epithelial cells and the pathogenesis of genetic disorders.

## Discussion

The phenotype similar to ODDD patients has not been adequately analyzed because Cx43-deficient mice are embryonic lethal (Reaume et al., 1995; Huang et al., 1998; Nagata et al., 2009). A mouse model of ODDD carrying a missense mutation in the *Cx43* gene (*Gja1Jrt/*+ mice) was isolated from an N-ethyl-N-nitrosourea mutagenesis screen (Flenniken et al., 2005). Unlike Cx43-deficient mice, this mutation is non-lethal, which allowed histological analysis of Cx43 in teeth. However, the molecular function of Cx43 in tooth development has not been sufficiently analyzed, although Cx43 dysfunction causes severe enamel hypoplasia and maxillofacial bone deformity. In our study, analysis using *Cx43*^−/−^ mice revealed decreased expression of Ambn during tooth development, revealing that this molecule is regulated by Cx43-mediated signaling. Enamel hypoplasia in ODDD patients and in Cx43 mutant mice likely arises, at least in part, due to reduced Ambn expression. The phenotype of the multilayer formation was similar to that found in the teeth of mice with Ambn deficiency (Fukumoto et al., 2004). Ambn is an enamel matrix protein secreted specifically by ameloblasts that functions as a cell adhesion molecule. *Ambn*^−/−^ mice display abnormal dental epithelium proliferation and multiple cell layers, and also develop severe enamel dysplasia and odontogenic tumors (Fukumoto et al., 2004). Furthermore, in *Ambn*^−/−^ mice, ameloblast polarization is inhibited during the secretory stage and cell proliferation of the inner enamel epithelium is increased. In fact, even in *Cx43*^−/−^ mice, ameloblast polarity was lost (Figure 3A). Further, the number of cells in the inner enamel epithelium, but not in the stratum intermedium, was increased (Figure 3B). Thus, the abnormal tooth phenotypes observed in *Cx43*^−/−^ mice were partly due to the reduced expression of enamel matrix proteins such as Ambn.

In dental epithelial cells, TGF-β1 and BMPs induce Ambn expression. TGF-β1 and BMP bind to their receptors and phosphorylate the downstream signaling molecules Smad and ERK1/2 (Guo and Wang, 2009; Luo, 2017). BMPs are important molecules for bone formation and are involved in the activation and induction of bone-specific transcription factors such as Runx2 (Komori et al., 1997; Chen et al., 2012). A Runx2 binding region is present in the promoter region of the *Ambn* gene (Dhamija and Krebsbach, 2001; Camilleri and McDonald, 2006; Kim et al., 2018), suggesting that TGF-β1 and BMP signaling are important for the expression of Ambn *via* Runx2. In fact, phosphorylation by ERK1/2 is required for the activation of Runx2, and phosphorylated Runx2 binds to Smad molecules to activate gene transcription (Lai and Cheng, 2002; Franceschi and Xiao, 2003; Chen et al., 2012). Both TGF-β1-overexpressing and -deficient mice have severe enamel hypoplasia (Haruyama et al., 2006; Song et al., 2018). These results indicate that the expression of TGF-β1 and its signaling pathway are important for enamel formation during tooth development. However, it is unclear how TGF-β1 and BMP signals are controlled by Cx43.

In osteoblastic ROS 17/2.8 cells, overexpressing the active forms of MEK, Raf, or Ras enhances the transcription of the osteocalcin gene through the connexin response element, whereas MEK and PI3K inhibitors repress transcription (Stains and Civitelli, 2005). In addition, this gene activation requires phosphorylation of the transcription factor Sp1 by ERK1/2. These results suggest that gap junctions, especially Cx43, may selectively regulate ERK1/2 phosphorylation induced by TGF-β1 in dental epithelial cells. Since Sp1 is expressed in almost all tissues, it is unlikely that it has a specific action during tooth development. Sp3 and Sp6/epiprofin are Sp family transcription factors that are involved in tooth development. The transcriptional activity of Ambn was induced by TGF-β, but not in the presence of oleamide (Figure 4C). TGF-β1-induced Ambn promoter activity was further enhanced by Sp6/epiprofin overexpression in dental epithelial cells. This facilitation was almost completely blocked by oleamide (data not shown), suggesting that the induction of Ambn expression by Sp6/epiprofin may require Sp6/epiprofin and a synergistic interaction with active Runx2. Alternatively, although Sp1 is phosphorylated by ERK1/2 and regulates osteocalcin transcription (Stains and Civitelli, 2005), Sp1 involvement has yet to be reported in tooth development. Enamel hypoplasia and decreased Ambn levels are observed in Sp3-deficient mice, similar to observations in Sp6/epiprofin-deficient mice (Bouwman et al., 2000; Nakamura et al., 2008). Since Sp3 expression was not affected by TGF-β1 stimulation or by gap junction inhibitors (data not shown), Sp3 may be ubiquitously expressed. Similar to Sp6/epiprofin, Sp3 may directly or indirectly regulate Ambn expression through ERK1/2, though this remains to be investigated.

The phosphorylation of ERK1/2 by Cx43 is important for Runx2-mediated transcriptional regulation of Ambn. However, it is unclear how Cx43 regulates ERK phosphorylation. Cx43 is required for growth factor-stimulated ERK1/2 phosphorylation; however, the mechanism by which Cx43 gap junction activity regulates ERK1/2 phosphorylation is not yet clear. Gap junction channels allow for the transfer of IP3 and Ca^2+^ between cells. The binding of IP3 to its receptors in the ER membrane promotes Ca^2+^ release from the ER (Mikoshiba, 2007), thereby increasing intracellular Ca^2+^. Further, Ca^2+^ efflux from the ER *via* the IP3R Ca^2+^ channel is essential for the conversion of Ras-GDP to its -GTP form, an upstream molecule of ERK1/2 (Pérez-Sala and Rebollo, 1999). We explored the involvement of the IP3/IP3R channel in the regulation of ERK1/2 phosphorylation through gap junctions by examining the effects of an IP3R agonist and antagonist on ERK1/2 phosphorylation. When dental epithelial cells were treated with the IP3R agonist, adenophostin-A, which stimulates the release of Ca^2+^ from the ER, ERK1/2 phosphorylation was induced without TGF-β1 stimulation. In contrast, treatment with the IP3R antagonist 2-APB inhibited TGF-β1-induced ERK1/2 phosphorylation (Figure 5F). These results suggest that IP3R is involved in ERK1/2 phosphorylation induced by TGF-β1.

An increase in intracellular calcium levels induced by Ca^2+^ ionophores reduces the permeability of Cx43 gap junctions (Lurtz and Louis, 2007). Decreased permeability through gap junctions is prevented by calmodulin inhibitors, but it is not affected by inhibitors of calmodulin-dependent protein kinase II or protein kinase C (Lurtz and Louis, 2007), which indicates that the interaction between intracellular Ca^2+^ and calmodulin plays an important role in Cx43 gating. Pretreatment with ionomycin prior to TGF-β1 stimulation suppressed ERK1/2 phosphorylation (data not shown). This may be because the Cx43 gates were closed by ionomycin. In any case, IP3R in the ER membrane is involved in ERK1/2 phosphorylation, and Cx43 may be involved in this process. When dental epithelial cells are stimulated with TGF-β1, the intracellular calcium concentration rapidly increases. This increase in intracellular calcium is triggered by IP3 binding to receptors on the ER membrane, which subsequently triggers calcium release from the ER. Calcium release from the ER was unchanged with or without Cx43 overexpression. Previously, we reported that gap junction proteins such as Panx3 may regulate calcium release from the ER (Ishikawa et al., 2011). Gap junctional proteins, but not Cx43 may regulates calcium release from ER membrane. However, a subsequent slow increase in intracellular calcium levels was observed in Cx43-overexpressing cells (Figure 6A). This enhanced intracellular calcium level was suppressed by the gap junction inhibitor 18α-GA (Figure 6C), and was also observed in cells in which siCx43 suppressed CX43 expression (Figure 6B). In contrast, intracellular calcium levels regulated by Cx43 were not inhibited by 2-APB, which inhibits calcium release from the ER. This result revealed that Cx43 regulates intracellular calcium levels *via* gap junctions rather than by calcium release from the ER. In fact, stimulation with TGF-β1 when cells are sparsely cultured does not adequately phosphorylate ERK1/2. Therefore, we believe that calcium influx through gap junctions may be important for ERK1/2 phosphorylation. Further, these results show that Cx43 may regulate ERK1/2 phosphorylation *via* intracellular calcium levels.

## Data Availability Statement

The original contributions presented in the study are publicly available. This data can be found here: https://www.ncbi.nlm.nih.gov/geo/GSE146855.

## Ethics Statement

The animal study was reviewed and approved by ethics committee of Kyushu University Animal Experiment Center.

## Author Contributions

AY and KY carried out experimental work, data analysis, interpretation, and writing of the manuscript. MI, KS, YC, RH, EF, SH, MC, TN, and TI contributed to experimental work. AY, KY, and SF carried out data interpretation and manuscript revision. AY and SF contributed to concept and design of research. All authors contributed to the article and approved the submitted version.

## Funding

This study was supported by a Grant-in-Aid from the Japan Society for the Promotion of Science (JSPS) KAKENHI (JP24390460 to AY, JP17H01606 and JP20K20612 to SF, and JP18H03012 to KY).

## Conflict of Interest

The authors declare that the research was conducted in the absence of any commercial or financial relationships that could be construed as a potential conflict of interest.

## Publisher’s Note

All claims expressed in this article are solely those of the authors and do not necessarily represent those of their affiliated organizations, or those of the publisher, the editors and the reviewers. Any product that may be evaluated in this article, or claim that may be made by its manufacturer, is not guaranteed or endorsed by the publisher.
